# A novel mutation in the major intrinsic protein (MIP) associated with autosomal dominant congenital cataracts in a Chinese family

**Published:** 2010-03-25

**Authors:** Wei Wang, Jin Jiang, Yanan Zhu, Jinyu Li, Chongfei Jin, Xingchao Shentu, Ke Yao

**Affiliations:** 1 Eye Center of the 2nd Affiliated Hospital, Medical College of Zhejiang University, Hangzhou, China; 2 Department of Ophthalmology, Zhejiang Provincial People’s Hospital, Hangzhou, China

## Abstract

**Purpose:**

To detect the underlying genetic defect in a Chinese family affected with bilateral congenital cataracts.

**Methods:**

A detailed family history and clinical data were recorded. Mutation screening was performed in the nuclear cataract-related gene by bidirectional sequencing of the amplified products. The mutation was verified by denaturing high-performance liquid chromatography (DHPLC).

**Results:**

Two cataract phenotypes were observed within this family: one eye exhibited Y-suture and nuclear pulverulent opacification of the lens, while the others exhibited complete opacification in the fetal nuclear region. Sequencing of the candidate genes detected a heterozygous c.319G>A change in the coding region of the major intrinsic protein (*MIP*), resulting in the substitution of a highly conserved Valine by Isoleucine (p.V107I).The mutation was confirmed by DHPLC.

**Conclusions:**

This study has identified a novel *MIP* mutation, p.V107I in a Chinese family with congenital cataracts. To the best of our knowledge, this is the first reported case of cataracts caused by a mutation in the second extracellular loop domain of MIP.

## Introduction

The ocular lens is a transparent organ that focuses light onto the retina. Cataracts, including spontaneous onset (congenital or juvenile) and age-related, are clouding of the clear lens, which can eventually lead to loss of vision. Although congenital cataract is less common than age-related cataract, it is the leading cause of visual disability in children [1]. Statistic analyses have revealed approximately one third of congenital cataracts to be familial [2], among which the autosomal dominant type is the most common mode of inheritance [3]. To date, autosomal dominant congenital cataracts (ADCC) have been linked to mutations of several different genes [4]. Two types of these genes were shown to be related to congenital nuclear cataracts: (1) Genes encoding crystallins: αA-crystallin (*CRYAA*) [5], βA1-crystallin (*CRYBA1*) [6], βB1-crystallin (*CRYBB1*) [7], βB2-crystallin (*CRYBB2*) [8], γC-crystallin (*CRYGC*) [9], and γD-crystallin (*CRYGD*) [10]; (2) Genes encoding membrane transport proteins: *GJA3* (Connexin46, Cx46) [11], *GJA8* (Connexin50, Cx50) [12], and *MIP* (major intrinsic protein or Aquaporin 0, AQP0) [13].

The connexins form gap junction channels for the intercellular transfer of small molecules and ions [14], while AQP0 functions as lens-specific water channel [15]. The lens, in the absence of a vascular supply, relies on metabolic co-operation through transmembrane channels, which have been proposed to supply deeper-lying fiber cells with nutrients and to clear waste products [14]. Therefore these channels formed by the connexins and AQP0 play essential roles in the maintenance of lifelong lens transparency.

In this study, we screened nuclear-cataract-associated genes by direct sequencing in a Chinese family with congenital Y-sutural and nuclear cataracts. In the first exon of *MIP*, We detected a novel G>A transition, which leads to a missense mutation in the extracellular space of this transmembrane protein.

## Methods

### Clinical evaluation and collection of genetic materials

A three-generation family with congenital cataracts was studied at the Eye Center of the 2nd Affiliated Hospital, Medical College of Zhejiang University, Hangzhou, China. Appropriate informed consent in accordance with the Declaration of Helsinki and the Zhejiang Institutional Review Board approval was obtained from all participants. Nine individuals from the family participated in the study, 4 affected and 5 unaffected individuals, of whom 3 were spouses. The status was determined by a history of cataract extraction or ophthalmologic examination, including visual acuity, slit lamp, and fundus examination. The phenotypes were documented by slit lamp photography. Blood samples were obtained by venipuncture, collected in a BD Vacutainer (BD, San Jose, CA) containing EDTA. Genomic DNA was extracted using the QIAmp Blood kit (Qiagen, Hilden, Germany) following the manufacturer’s instructions

### PCR and DNA sequencing

Mutation screening was performed in the exon regions of the following candidate genes: *CRYAA, CRYBA1, CRYBB1, CRYBB2, CRYGC*, *CRYGD*, *GJA3, GJA8*, and *MIP.* The coding regions were amplified using previously published primer sequences [5,6,8,10,16,17]. PCR reactions were performed under the following conditions: 95 °C preactivation for 5 min, 10 cycles of touchdown PCR with 0.5 °C down per cycle from 60 °C to 55 °C, followed by 25 cycles with denaturation at 95 °C for 45 s, annealing at 55 °C for 45 s and extension at 72 °C for 45 s. PCR products were isolated by electrophoresis on 3% agarose gels and sequenced using the BigDye Terminator Cycle sequencing kit V 3.1 (ABI Applied Biosystems, Sangon Co, Shanghai, China) on an ABI PRISM 3730 Sequence Analyzer (ABI), according to the manufacturer’s directions

### Denaturing high-performance liquid chromatography

Denaturing high performance liquid chromatography (DHPLC) was used to screen exon 1 of *MIP* in the affected patients, other family members, and 100 control subjects using a commercial system (Wave DHPLC; Transgenomic, San Jose, CA) .The conditions were as follows: initial concentration at 44% of buffer A (0.1 M triethylammonium acetate, TEAA; Transgenomic) and 56% of buffer B (0.1 M TEAA containing 25% acetonitrile; Transgenomic) at 61.0 °C

## Results

### Clinical evaluation

**Figure 1:**
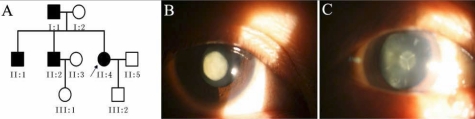
Cataracts in the family. **A**: Pedigree of the family with autosomal dominant cataract. The black arrow indicates the proband. **B**: A slit lamp photograph shows complete opacification of the lens fetal nucleus in the proband (II:4). **C**: A slit lamp photograph of the right eye lens of the proband’s father (I:1) reveals the Y sutural and nuclear pulverulent cataract.

We identified a three-generation family with autosomal dominant congenital cataracts (Figure 1A). Opacification of the lens was bilateral in all of the affected individuals. There was no family history of other ocular or systemic abnormalities. The patients’ visual acuity ranged from finger count to 20/40 in the unoperated eyes. The impaired vision had been present since childhood and no complaint of decreased visual acuity with age from any of the patients. The proband was a 39-year old female (II:4). All of the affected individuals, except for individual I:1, exhibited complete opacification of the fetal nucleus (Figure 1B). However, the cataracts of individual I:1 were very special: the left eye lens appeared to have complete opacification of the fetal nuclear region, while the right eye lens exhibited Y-suture opacification surrounded by a nuclear pulverulent cataract (Figure 1C) .

### Mutation detection

**Figure 2:**
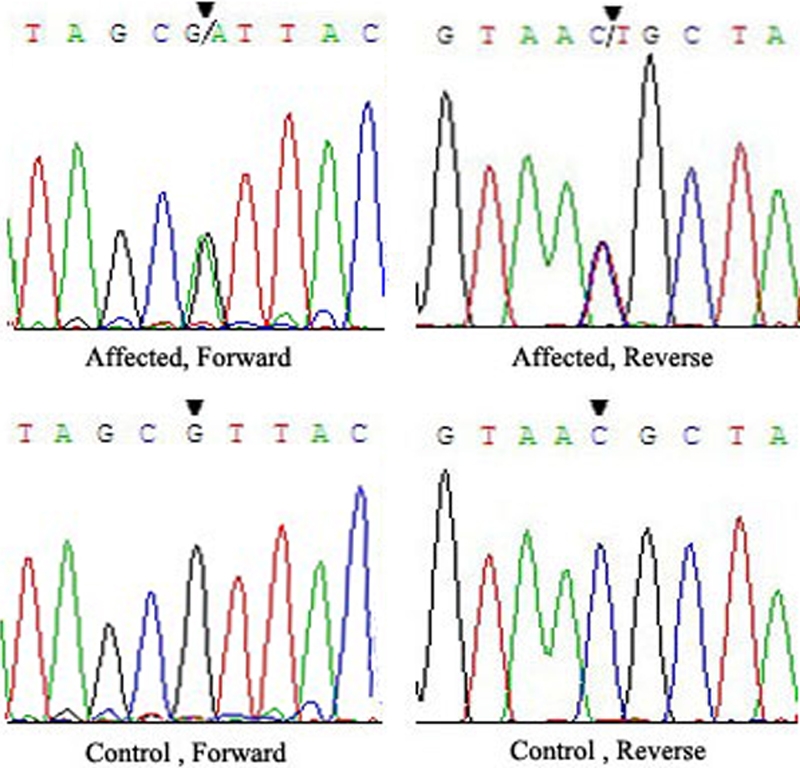
Forward and reverse sequence analysis of the affected and unaffected individuals in this ADCC Chinese family. A heterozygous mutation (c.319G>A) in exon 1 of *MIP* (black triangles) is shown.

Bidirectional sequencing of the coding regions of the candidate genes revealed a heterozygous change, G>A, at position 319 (c.319G>A) in *MIP* in all of the affected individuals (Figure 2), resulting in the replacement of highly conserved Valine by Isoleucine (p.V107I).

### DHPLC analysis

**Figure 3:**
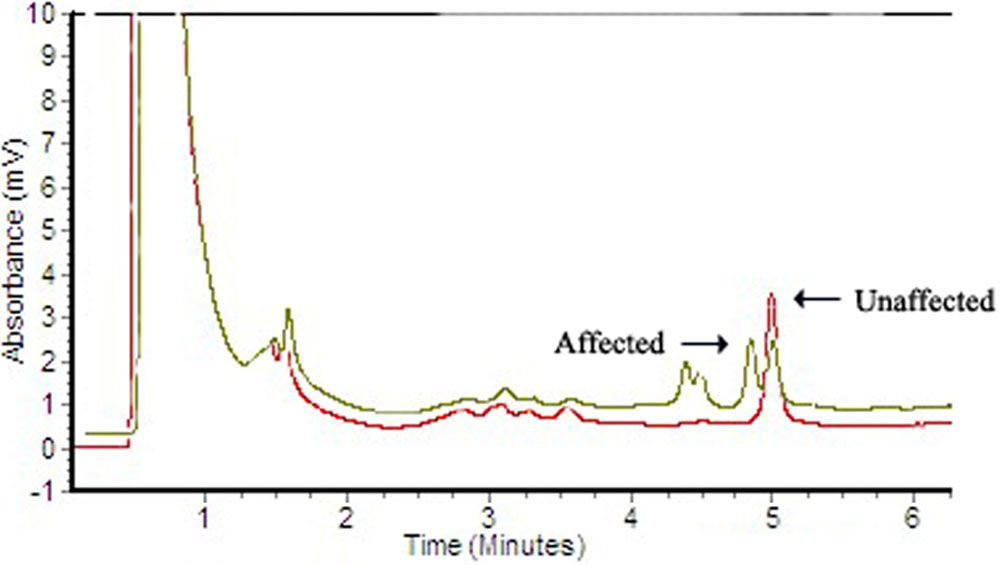
Denaturing high-performance liquid chromatography results of wild-type and mutated *MIP*. The DHPLC results shows variant traces for *MIP* compared to the wild-type (WT) trace. Profiles in brown contain the mutation; the profile in red is the wild type.

DHPLC confirmed this mutation, which cosegregated in all the affected individuals in the family, and this mutation was not observed in any of the unaffected family members or 100 unrelated control individuals (Figure 3).

### Multiple-sequence alignment and mutation analysis

**Figure 4:**
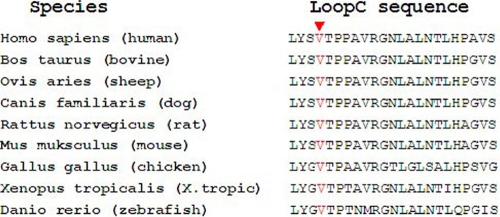
Multiple-sequence alignment of MIP from different species. Multiple-sequence alignment of the different species reveals that codon 107, where the mutation (p.V107I) occurred, is highly conserved.

Using the NCBI website, we obtained the multiple sequence alignment of the AQP0 protein in various species including *Homo sapiens* (NP_036196.1), *Bos taurus* (NP_776362.1), *Ovis aries* (NP_001153230.1), *Canis familiaris* (NP_001074369.1), *Rattus norvegicus* (NP_001099189.1), *Mus musculus* (NP_032626.2), *Gallus gallus* (NP_989597.1), *Xenopus tropicalis* (NP_001090816.1) and *Danio rerio* (NP_001003534.1). We found that codon 107, where the mutation (p.V107I) occurred, was phylogenetically conserved (Figure 4).

## Discussion

**Figure 5:**
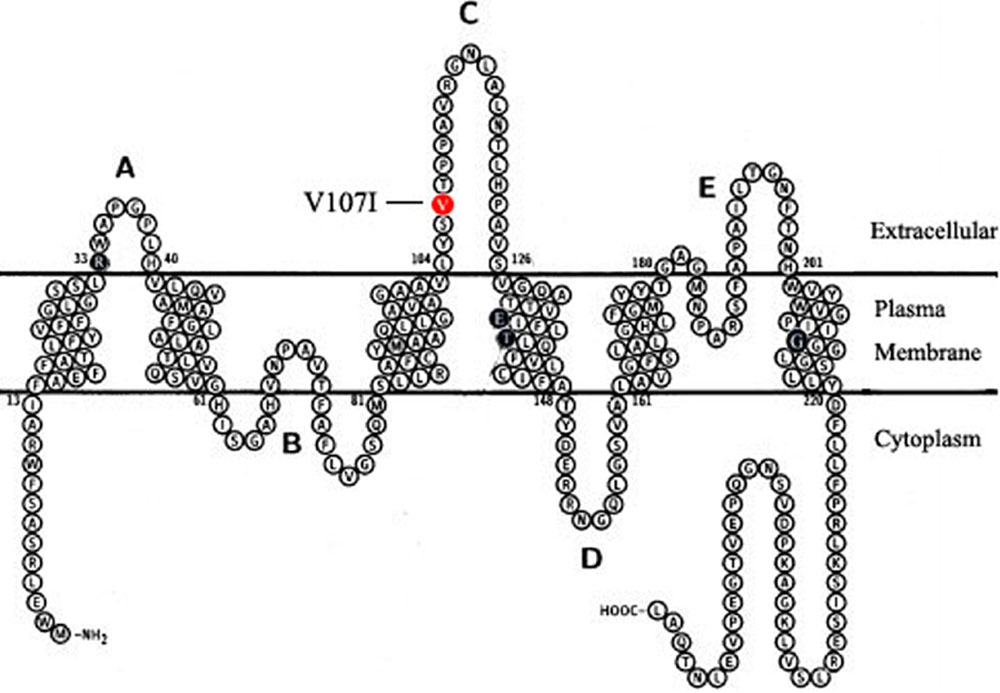
Topology of AQP0 and mutation analysis. Four reported missense mutations are marked in black: R33C, E134G, T138R, R233K. The novel mutation, V107I, is marked in red. This mutation located at the extracellular loopC of AQP0. (Modified from [36], permission granted by Prof. Peter Agre).

AQP0 is a member of the aquaporin family, which forms pores that are either highly selective for water or permeable to other small neutral solutes, such as glycerol and urea [18]. As the water pores confer rapid movements of water across the plasma membranes, they are considered essential for the lens microcirculation system. Besides the water permeability function, AQP0 sometimes plays a cell-to-cell adhesion role [19,20], that helps compact the highly ordered fiber cells, thus minimizing extracellular space and light scattering. Moreover, AQP0 has been reported to interact with many other lens components including crystallins, lipids, and cytoskeletal proteins. These interactions were demonstrated to be important in maintaining the lens structure and homeostasis [21-23]. Mutation in *MIP* in humans and mice has been reported to induce genetic cataracts in various studies [24-28]. To date, several mutations in human *MIP* have been identified, most of which are missense mutations (Figure 5).The cataracts induced by MIP mutation are usually located in the lens nuclear region (Table 1).

**Table 1:** Previously reported mutations in MIP gene associated with cataracts

**DNA change**	**Protein change**	**Mode of inheritance**	**Location/ MIP domain**	**Phenotype description**	**Origin of family**	**References**
c. A401G	E134G	AD	H4 TMα-helics	Lamellar and sutural	English	[24]
c.C413G	T138R	AD	H4 TMα-helics	Polymorphic	English	[24]
A deleted G at nt.3223	Delete mutation at codon 213	AD	H6 TMα-helics	Radiating, vacuolar, or dense embryonal nuclear	American	[25]
c.G698A	R233K	AD	COOH-terminus	Posterior of the right, anterior polar in the left	Chinese	[27]
c.C97T	R33C	AD	Loop A	Total	Chinese	[26]
IVS3–1 G>A		AD	COOH-terminus	Snail-like	Chinese	[13]

*MIP* expression starts as soon as the first primary fibers begin filling the lens vesicle, and continues to be expressed as the secondary fibers are differentiated from the equatorial epithelial cells [29]. When the terminal ends of the secondary fibers abut each other, lens sutures form [30]. Deletion of the *MIP* gene in mice leads to a lack of suture formation, resulting in the perturbed accommodation and focusing properties of the ocular lens [31]. These data highlight the important role that AQP0 plays in fiber adhesion and suture formation. The special cataract phenotype observed in this family, Y suture and nuclear opacities, is consistent with the localization and function of the AQP0 protein. Abnormal development of sutures has been reported in association with specific types of cataracts. Sutural cataract may occur as either an isolated type of cataract [32] or in association with opacities involving other lens regions [33,34]. In this family, Y suture and nuclear pulverulent opacities were observed in one affected individual, while the others were total opacification in the fetal nuclear region, including the Y suture. The diverse cataract phenotypes caused by the same *MIP* gene suggests that other genetic modifiers are likely to influence the expression and function of AQP0 in lens development and Y suture formation.

Structural studies have shown that the transmembrane (TM) AQP0 contains two tandem repeats, each of which has three TMα-helics and a hydrophobic loop with a conserved asparagine- proline-alanine (NPA) motif. Six TM domains are connected by 5 loops, three of which are extracellular (loop A, C, E), while the others are intracellular (loop B, D) [35]. The extracellular loopC, which connects the third and forth helices, mediates most of the junction-forming interactions of AQP0. The novel c.319G>A transition results in the substitution of a Valine for an Isoleucine (p.V107I), in loopC of AQP0 (Figure 5). The high conservation of V107 from zebrafish to humans indicates the importance of this residue.

There has been little functional investigation of the mutant AQP0 protein. Research on a *Xenopus* oocyte expression system showed that both the E134G and T138R mutations result in the loss of membrane water channel activity [36]. The deletion mutation at codon 213 created a frame-shift, and was demonstrated to interfere with the permeability of the water channel and the trafficking of proteins, thus leading to cataracts [37]. Electron crystallography research on the core of the sheep lens showed that the junction formation contacts are formed by the residues in loopC, including Pro109 and Pro110, and Arg113 and Pro123 [38]. Therefore, the Valine at site 107 may lie close enough to participate in or stabilize the contacting junction. Given that the mutation affects the connections of the neighbor helices, it is likely that it affects the tight gap junction formation by affecting the adhesion and regulation of the extracellular space volume. Water channel gating and the microcirculation may also be interrupted due to the improper organization of fibers. Further investigation is required to confirm that this is the mechanism by which the mutation affects the protein.

In summary, we identified a novel mutation of the human *MIP* gene segregated with Y sutural and nuclear cataract. This expands the spectrum of *MIP* mutations causing autosomal dominant nuclear cataract, both in terms of ethnicity and in terms of the location of the mutation in the loopC region of the protein.

## References

[1] Hejtmancik JF Smaoui N Molecular genetics of cataract. Dev Ophthalmol 2003 37 67 82 12876830 10.1159/000072039

[2] Amaya L Taylor D Russell-Eggitt I Nischal KK Lengyel D The morphology and natural history of childhood cataracts. Surv Ophthalmol 2003 48 125 44 12686301 10.1016/s0039-6257(02)00462-9

[3] Vanita Singh D Genetic and segregation analysis of congenital cataract in the Indian population. Clin Genet 1999 56 389 93 10668929 10.1034/j.1399-0004.1999.560507.x

[4] Reddy MA Francis PJ Berry V Bhattacharya SS Moore AT Molecular genetic basis of inherited cataract and associated phenotypes. Surv Ophthalmol 2004 49 300 15 15110667 10.1016/j.survophthal.2004.02.013

[5] Vanita V Singh JR Hejtmancik JF Nuernberg P Hennies HC Singh D Sperling K A novel fan-shaped cataract-microcornea syndrome caused by a mutation of CRYAA in an Indian family. Mol Vis 2006 12 518 22 16735993

[6] Lu S Zhao C Jiao H Kere J Tang X Zhao F Zhang X Zhao K Larsson C Two Chinese families with pulverulent congenital cataracts and deltaG91 CRYBA1 mutations. Mol Vis 2007 13 1154 60 17653060

[7] Meyer E Rahman F Owens J Pasha S Morgan NV Trembath RC Stone EM Moore AT Maher ER Initiation codon mutation in betaB1-crystallin (CRYBB1) associated with autosomal recessive nuclear pulverulent cataract. Mol Vis 2009 15 1014 9 19461930 PMC2684559

[8] Litt M Carrero-Valenzuela R LaMorticella DM Schultz DW Mitchell TN Kramer P Maumenee IH Autosomal dominant cerulean cataract is associated with a chain termination mutation in the human beta-crystallin gene CRYBB2. Hum Mol Genet 1997 6 665 8 9158139 10.1093/hmg/6.5.665

[9] Yao K Jin C Zhu N Wang W Wu R Jiang J Shentu X A nonsense mutation in CRYGC associated with autosomal dominant congenital nuclear cataract in a Chinese family. Mol Vis 2008 14 1272 6 18618005 PMC2447816

[10] Zhang LY Yam GH Fan DS Tam PO Lam DS Pang CP A novel deletion variant of gammaD-crystallin responsible for congenital nuclear cataract. Mol Vis 2007 13 2096 104 18079686

[11] Burdon KP Wirth MG Mackey DA Russell-Eggitt IM Craig JE Elder JE Dickinson JL Sale MM A novel mutation in the Connexin 46 gene causes autosomal dominant congenital cataract with incomplete penetrance. J Med Genet 2004 41 e106 15286166 10.1136/jmg.2004.018333 PMC1735867

[12] Arora A Minogue PJ Liu X Addison PK Russel-Eggitt I Webster AR Hunt DM Ebihara L Beyer EC Berthoud VM Moore AT A novel connexin50 mutation associated with congenital nuclear pulverulent cataracts. J Med Genet 2008 45 155 60 18006672 10.1136/jmg.2007.051029 PMC2756454

[13] Jiang J Jin C Wang W Tang X Shentu X Wu R Wang Y Xia K Yao K Identification of a novel splice-site mutation in MIP in a Chinese congenital cataract family. Mol Vis 2009 15 38 44 19137077 PMC2615830

[14] Gong X Cheng C Xia CH Connexins in lens development and cataractogenesis. J Membr Biol 2007 218 9 12 17578632 10.1007/s00232-007-9033-0

[15] Han BG Guliaev AB Walian PJ Jap BK Water transport in AQP0 aquaporin: molecular dynamics studies. J Mol Biol 2006 360 285 96 16756992 10.1016/j.jmb.2006.04.039

[16] Hansen L Yao W Eiberg H Funding M Riise R Kjaer KW Hejtmancik JF Rosenberg T The congenital “ant-egg” cataract phenotype is caused by a missense mutation in connexin46. Mol Vis 2006 12 1033 9 16971895

[17] Schmidt W Klopp N Illig T Graw J A novel GJA8 mutation causing a recessive triangular cataract. Mol Vis 2008 14 851 6 18483562 PMC2375854

[18] Verkman AS Role of aquaporin water channels in eye function. Exp Eye Res 2003 76 137 43 12565800 10.1016/s0014-4835(02)00303-2

[19] Gonen T Cheng Y Kistler J Walz T Aquaporin-0 membrane junctions form upon proteolytic cleavage. J Mol Biol 2004 342 1337 45 15351655 10.1016/j.jmb.2004.07.076

[20] Kumari SS Varadaraj K I ntact AQP0 performs cell-to-cell adhesion. Biochem Biophys Res Commun 2009 390 1034 9 19857466 10.1016/j.bbrc.2009.10.103 PMC2892625

[21] Yu XS Jiang JX Interaction of major intrinsic protein (aquaporin-0) with fiber connexins in lens development. J Cell Sci 2004 117 871 80 14762116 10.1242/jcs.00945

[22] Fan J Fariss RN Purkiss AG Slingsby C Sandilands A Quinlan R Wistow G Chepelinsky AB Specific interaction between lens MIP/Aquaporin-0 and two members of the gamma-crystallin family. Mol Vis 2005 11 76 87 15692460

[23] Gonen T Cheng Y Sliz P Hiroaki Y Fujiyoshi Y Harrison SC Walz T Lipid-protein interactions in double-layered two-dimensional AQP0 crystals. Nature 2005 438 633 8 16319884 10.1038/nature04321 PMC1350984

[24] Berry V Francis P Kaushal S Moore A Bhattacharya S Missense mutations in MIP underlie autosomal dominant 'polymorphic' and lamellar cataracts linked to 12q. Nat Genet 2000 25 15 7 10802646 10.1038/75538

[25] Geyer DD Spence MA Johannes M Flodman P Clancy KP Berry R Sparkes RS Jonsen MD Isenberg SJ Bateman JB Novel single-base deletional mutation in major intrinsic protein (MIP) in autosomal dominant cataract. Am J Ophthalmol 2006 141 761 3 16564824 10.1016/j.ajo.2005.11.008 PMC1463993

[26] Gu F Zhai H Li D Zhao L Li C Huang S Ma X A novel mutation in major intrinsic protein of the lens gene (MIP) underlies autosomal dominant cataract in a Chinese family. Mol Vis 2007 13 1651 6 17893667

[27] Lin H Hejtmancik JF Qi Y A substitution of arginine to lysine at the COOH-terminus of MIP caused a different binocular phenotype in a congenital cataract family. Mol Vis 2007 13 1822 7 17960133

[28] Shiels A Bassnett S Mutations in the founder of the MIP gene family underlie cataract development in the mouse. Nat Genet 1996 12 212 5 8563764 10.1038/ng0296-212

[29] Varadaraj K Kumari SS Mathias RT Functional expression of aquaporins in embryonic, postnatal, and adult mouse lenses. Dev Dyn 2007 236 1319 28 17377981 10.1002/dvdy.21125 PMC2534140

[30] Kuszak JR Zoltoski RK Tiedemann CE Development of lens sutures. Int J Dev Biol 2004 48 889 902 15558480 10.1387/ijdb.041880jk

[31] Al-Ghoul KJ Kirk T Kuszak AJ Zoltoski RK Shiels A Kuszak JR Lens structure in MIP-deficient mice. Anat Rec A Discov Mol Cell Evol Biol 2003 273 714 30 12845708 10.1002/ar.a.10080

[32] Vanita V Hejtmancik JF Hennies HC Guleria K Nurnberg P Singh D Sperling K Singh JR Sutural cataract associated with a mutation in the ferritin light chain gene (FTL) in a family of Indian origin. Mol Vis 2006 12 93 9 16518306

[33] Vanita Sarhadi VK Singh D Reis A Rueschendorf F Becker-Follmann J Jung M Sperling K A novel form of “central pouchlike” cataract, with sutural opacities, maps to chromosome 15q21–22. Am J Hum Genet 2001 68 509 14 11133359 10.1086/318189 PMC1235284

[34] Vanita Jung M Singh D Sperling K Singh JR Burger J A unique form of autosomal dominant cataract explained by gene conversion between beta-crystallin B2 and its pseudogene. J Med Genet 2001 38 392 6 11424921 10.1136/jmg.38.6.392 PMC1734905

[35] Chepelinsky AB Structural function of MIP/aquaporin 0 in the eye lens; genetic defects lead to congenital inherited cataracts. Handb Exp Pharmacol 2009 190 265 97 19096783 10.1007/978-3-540-79885-9_14

[36] Francis P Chung JJ Yasui M Berry V Moore A Wyatt MK Wistow G Bhattacharya SS Agre P Functional impairment of lens aquaporin in two families with dominantly inherited cataracts. Hum Mol Genet 2000 9 2329 34 11001937 10.1093/oxfordjournals.hmg.a018925

[37] Varadaraj K Kumari SS Patil R Wax MB Mathias RT Functional characterization of a human aquaporin 0 mutation that leads to a congenital dominant lens cataract. Exp Eye Res 2008 87 9 21 18501347 10.1016/j.exer.2008.04.001 PMC2504491

[38] Gonen T Sliz P Kistler J Cheng Y Walz T Aquaporin-0 membrane junctions reveal the structure of a closed water pore. Nature 2004 429 193 7 15141214 10.1038/nature02503

